# Plummer's Pill: Its Insolubility

**Published:** 1907-05-25

**Authors:** 


### Points in Treatment.

### PLUMMER'S PILL: ITS INSOLUBILITY.

Some interesting experiments have been made in the laboratories of Messrs. Southall Bros. and Barclay, at the instigation of Sir James Sawyer, upon the solubility of Plummer's pill as made according to the directions of the British Pharmacopœia. The results point to the necessity for modifying the formula for the pill, in order to make it more readily disintegrated in the alimentary canal. Plummer's pill is a remedy which is so frequently employed, and with such benefit in some cases, that it is well worth while trying to make sure that it does not pass through the body completely unchanged, as too often happens. It is useful in many nervous conditions, whether functional or organic; in those indefinite conditions which are popularly called "biliousness"; and in those still more indefinite cases in which that obscure effect is desired to which the term "alterative" is applied.

Plummer's pill, which is also called compound calomel pill, or the pilula hydrargyri subchloridi composita of the British Pharmacopœia, is made from calomel, sulphurated antimony, guaiacum resin, castor oil, and alcohol (90 per cent.). It contains 1 grain of calomel, 1 grain of sulphurated antimony, and 2 grains of guaiacum resin in 4½ grains.

Various samples of the pill, some fresh, some old, some coated, and some uncoated, were all examined under the same conditions by treatment with water for 48 hours with frequent agitation, the water being maintained at a temperature of 38° C., *i.e.*, about body temperature. In almost all cases the pills were just the same at the end of the experiment as at the beginning; in only a few instances did any disintegration of the pill occur, notwithstanding the fact that the submersion and agitation in warm water extended over a much longer period than would be the case in the alimentary canal. It is true that the hydrochloric acid in the gastric juice, and the alkali in the intestinal juices, acting successively upon the pill, might be thought likely to accelerate the disintegration; but that this is often not the case is shown by finding the pills unaltered in the stools.

The fault lies, not in the calomel nor in the sulphurated antimony, but in the guaiacum resin, castor oil, and alcohol; the interaction of these three substances seems to lead to the production of a very tenacious and insoluble product, and it is the latter which prevents the disintegration of the pill. Experiments were carried out to find a remedy for this. Sir James Sawyer points out that omission of the castor oil, as proposed by the Committee of Reference in Pharmacy, does not correct the defect. Pills made according to the pharmacopœial formula, but leaving out the castor oil, show little or no improvement in their power of disintegration when agitated in water at body temperature for two days. The oil may well be left out, but it is necessary to leave out the spirit also. Sir James Sawyer has proposed a formula for the pill in which syrupus glucosi is the only excipient. The prescription may be written with syrup of glucose in the place of the castor oil and the alcohol of the official formula; the result is a good pill, which disintegrates rapidly in water at body temperature, does not pass unaltered in the stools, and is likely, therefore, to be of much greater service as a therapeutic agent than is the usual Plummer's pill.

